# Extraction of Yttrium from Waste: Analysis of Hydrometallurgical Processing by Organic Acids and Life Cycle Assessment

**DOI:** 10.1021/acsomega.4c09774

**Published:** 2025-11-27

**Authors:** Luan Matheus da Silva Alvarenga, Mentore Vaccari, Denise Crocce Romano Espinosa, Amilton Barbosa Botelho Junior

**Affiliations:** † Department of Chemical Engineering, Polytechnic School, University of São Paulo, São Paulo 01246-904, Brazil; ‡ Department Civil, Environmental, Architectural Engineering and Mathematics, University of Brescia, Brescia 25121, Italy; § Department of Materials Science and Engineering, 2167 Massachusetts Institute of Technology, Cambridge, Massachusetts 02139, United States; ∥ Department of Chemical Engineering, Norwegian University of Science and Technology, Trondheim 7491, Norway

## Abstract

The increasing demand for rare earth elements has driven the search for efficient and sustainable recovery methods. Obsolete fluorescent lamps represent a significant secondary source of Y that can greatly contribute to the circular economy and the preservation of natural resources. With the gradual depletion of primary Y reserves and the rise in e-waste generation, the development of eco-friendly and economically feasible recovery techniques has become crucial. Additionally, strict legislation regarding the disposal of e-waste strengthens the need to improve recycling processes. This study aims to investigate the leaching of Y from obsolete fluorescent lamps by organic (C_6_H_8_O_7_, C_2_H_4_O_2_, and C_2_H_5_NO_2_) and inorganic acids (HNO_3_). We also seek to assess the environmental impact of this process through life cycle assessment (LCA). Leaching steps were performed with different acid concentrations, followed by selective precipitation with C_2_H_2_O_4_ for Y recovery. LCA was applied to evaluate the environmental impacts and identify critical points in the process. A high Y recovery rate (78.8% for C_2_H_5_NO_2_, 86.7% for C_6_H_8_O_7_, 100% for C_2_H_4_O_2_, and 95% for HNO_3_) was obtained with precipitation (C_2_H_5_NO_2_ and C_6_H_8_O_7_ liquor). Our environmental assessment revealed that leaching with organic acids presents a higher environmental impact due to production and disposal methods. The study demonstrated that it is possible to leach Y efficiently from fluorescent lamps using organic acids. The process may be a feasible alternative for large-scale Y recovery, improving organic acid production, and contributing to the sustainable recycling of e-waste and promoting the circular economy.

## Graphical abstract

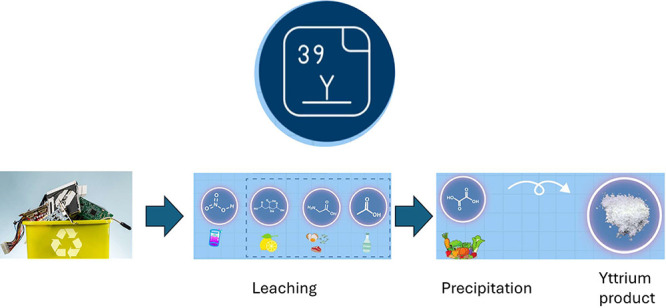

## 1 Introduction

Yttrium (Y) is a rare earth element with vast technological applications, particularly in the production of phosphors for electronic display screens.^1,2^ It is also widely used in lasers, superconductors, and high-performance ceramic materials.^3^ These technologies are important for the green energy transition, as fluorescent lamps and LEDs.^4–6^ Its production is performed from primary sources by mining, while the recycling rate from wastes (mainly e-waste) is low (3%).^3,4^ Production of this equipment tends to increase in the next years, raising also the production of Y and other rare earth elements.^3,7^ China is the main producer of rare earth elements (including Y), leading with 95% of worldwide production.^3,4^ As current practices have a large negative impact on the environment,^6^ we need to investigate and propose new technologies for the extraction of these elements.

The advantage of recycling is beyond the promotion of the circular economy, but meets the aims of urban mining^8^ and sustainable mining.^9^ Instead of extracting rare earth elements from mining only, urban mining transforms urban solid waste (as e-wastes) into a source of these elements and can be produced in any country/region where this waste is generated. It reduces dependence on the mining of specific natural resources.^3^ It is urgent to transform chemical processes into greener and economically feasible ones, and to achieve this goal, we aimed in this study to propose organic acids to obtain Y by hydrometallurgical process recycling. Literature reports the use of inorganic acids, such as H_2_SO_4_, HNO_3_, and HCl, which are produced from nonsustainable sources.^10^ Organic acids have come as a potential alternative for sustainable leaching.^11,12^

For our study, we used spent fluorescent lamp powder rich in Y to demonstrate the applicability of organic acid leaching. Amounts of Y can be similar to natural mining ores or even higher^12^; however, it may contain lower concentrations of other elements, while fluorescent lamps that are composed of Ca sulfate and silica^13^ or in LEDs containing Ga, Al, and other rare earth elements.^5^ It highlights recycling as an alternative for Y recovery from e-wastes.

In this study, acids such as oxalic acid (C_2_H_2_O_4_), citric acid (C_6_H_8_O_7_), acetic acid (CH_3_COOH), and glycine (C_2_H_5_NO_2_) were explored due to their ability to complex metal ions, enabling selective extraction. Nitric acid (HNO_3_), on the other hand, is known for its high efficiency, and we reported its use for comparison with the efficiency of organic acids. Organic acids can also be used as a precipitant agent after leaching. Martins et al. (2023) used C_2_H_2_O_4_ for Co precipitation after leaching of spent Li-ion batteries by C_6_H_8_O_7_, demonstrating that even a process combining organic acids in two different steps (leaching followed by precipitation) can be feasible. Oxalic acid (C_2_H_2_O_4_) and sodium oxalate (Na_2_C_2_O_4_) were tested for their roles in the precipitation step, forming insoluble compounds with Y and allowing its selective separation from other metals in the leach solution.

Beyond technical feasibility, a life cycle assessment (LCA) was performed to analyze the environmental impact of the recycling route using the current production path of organic acid production in different steps (leaching and purification). Additionally, the LCA provided insights into key environmental indicators such as greenhouse gas emissions, energy consumption, and resource depletion.^6,14^ By evaluating these metrics across the various stages of the recycling process, including transportation, chemical usage, and waste generation, we aimed to identify potential environmental benefits and challenges in the flowchart proposal.^10^ This comprehensive approach ensures that the recycling method not only achieves material recovery but also aligns sustainability goals by minimizing its ecological footprint.^12^

## 2 Materials and Methods

### 2.1 Characterization

The spent fluorescent sample was pretreated before experiments and obtained in a powder form by dismantling and comminution, and the characterization is reported elsewhere.^13^ Here, the goal was to quantify the elemental composition of the residue for mass balance and mineral assessment in leaching using organic acids. Chemical characterization was carried out by aqua regia (acid digestion for chemical dissolution) at a solid/liquid ratio of 1/40 in a beaker at 70 °C for 48 h under magnetic stirring. The liquor was analyzed by X-ray fluorescence spectroscopy (EDXRF, EDX-7200 Shimadzu) and wavelength-dispersive X-ray fluorescence spectroscopy (WDXRF, Shimadzu LAB CENTER XRF-1800).

The particle size distribution was evaluated using a Malvern Mastersizer 2000, and X-ray diffraction (XRD, MiniFlex 300 Rigaku) analysis was carried out to identify the main phases in the powder after drying at 60 °C for 24 h. The same procedure for characterization was also used for solids after leaching and precipitation experiments.

For EDXRF and WDXRF, the leach solution was placed in a sample holder for analysis using a Y calibration curve previously prepared with a standard solution diluted in 3% HNO_3_. For particle size analysis, the lamp powder was mixed with ultrapure water in a container under agitation and ultrasound to break up any agglomerations, and then passed through the Malvern. Finally, for XRD, the lamp powder was properly placed in the sample holder and analyzed from 20° to 80° with a step of 0.02°/s.

### 2.2 Acid Leaching Experiments

Leaching experiments were carried out with HNO_3_, C_6_H_8_O_7_, C_2_H_5_NO_2_, and C_2_H_4_O_2_ prepared with ultrapure water. The pH values of glycine (pH 2.0) and C_2_H_4_O_2_ (pH 0) were adjusted by adding HNO_3_. The parameters studied were S/L ratio (1/5–1/20), concentration (0.5–4 mol/L), temperature (25–90 °C), time (30–360 min), and pH (0–7) (Table S1 Supporting Information). These experiments were carried out aiming at the selective extraction of Y from the spent powder and comparing the efficiency among organic acids. These organic acids were chosen due to their potential production under the biotechnological approach, with lower environmental and economic impact than inorganic acids, and these differences were also evaluated in the LCA calculation. HNO_3_ leaching was used for comparison with organic acids on Y leaching. The use of H_2_SO_4_ is largely reported in the literature and was compared with our results.^13^

Organic complexes formed in the solid residue between acid and Y were analyzed by FT-IR (Shimadzu, Xross over). The precipitate was dried at 25 °C for 24 h and placed directly into an attenuated total reflectance (ATR = Bruker Tensor27) accessory. The FTIR was then calibrated with an acetone reading, and the background spectrum was recorded to eliminate interferences (e.g., water vapor and carbon dioxide). The spectrum of the sample reflects the absorption bands of the functional groups present in the material.

Leaching experiments were performed in a three-necked bottle reactor connected to a condenser with water circulating at 12 °C, another was sealed, and the last one was connected to a thermometer. The solution was heated to the desired temperature under magnetic stirring, and the sample was added to the reactor as the temperature was reached. After the reaction time, the leach solution was vacuum filtered (3 μm), and the solid (leaching residue) was washed with ultrapure water. The leaching solution was analyzed by EDXRF, and the leaching residue was analyzed by WDXRF and XRD for mass balance. The solid was prepared for XRD analysis (dried and macerated until it turned into powder), and the sample was placed in the sample holder for the analysis.

### 2.3 Precipitation

In this section, we aimed to evaluate the impact of the acid matrix (organic or inorganic) on Y precipitation as oxalate. Precipitation experiments were carried out with C_2_H_2_O_4_ and Na_2_C_2_O_4_, varying stoichiometry proportion (0, 25, 50, 75, and 100% of excess) to precipitate as Y_2_(C_2_O_4_)_3_ (eq 1). The overall extraction efficiency was then calculated by mass balance, and the data were used for LCA.

$$2Y_{\left(\mathrm{aq}\right)}^{3+}+3(\mathrm{Na}\;\mathrm{or}\;H{)}_{2}C_{2}{O_{4}}_{\left(s\right)}\to Y_{2}(C_{2}O_{4}{{)}_{3}}_{\left(s\right)}+6(\mathrm{Na}\;\mathrm{or}\;H{)}_{\left(\mathrm{aq}\right)}^{+}$$ 1

Experiments were carried out with 0.1 L of real leaching solution in a beaker under magnetic stirring for 30 min, where the precipitant agent was added as a solid while the solution was mixed. After the reaction time (30 min), the mixture was filtered as in leaching experiments. All leach solutions (inorganic and organic acids) were evaluated. The solution after precipitation was analyzed by EDXRF. The solid obtained was weighed and analyzed by FTIR and XRD after drying at 60 °C for 24 h. Calcination to obtain Y_2_O_3_ was also performed and analyzed. Chemical analysis was carried out, as in the characterization step, to determine the purity.

### 2.4 Life-Cycle Assessment

LCA serves as a method for evaluating the environmental or potential environmental impacts throughout a product’s life cycle.^10^ The main research question for our study was a comparison of organic and inorganic acids for the extraction of Y from waste. The modeling and simulation processes were evaluated by using SimaPro 8 software with the EcoInvent 3.1 database. Our analysis was based on the flowchart proposed from lab experiments (Figure 1a), and the system boundary was established (Figure 1b) by mass and energy balance. Simplified LCA was conducted for the leaching experiments, considering transport and energy consumption to produce and dispose of the acids.

**Figure 1:**
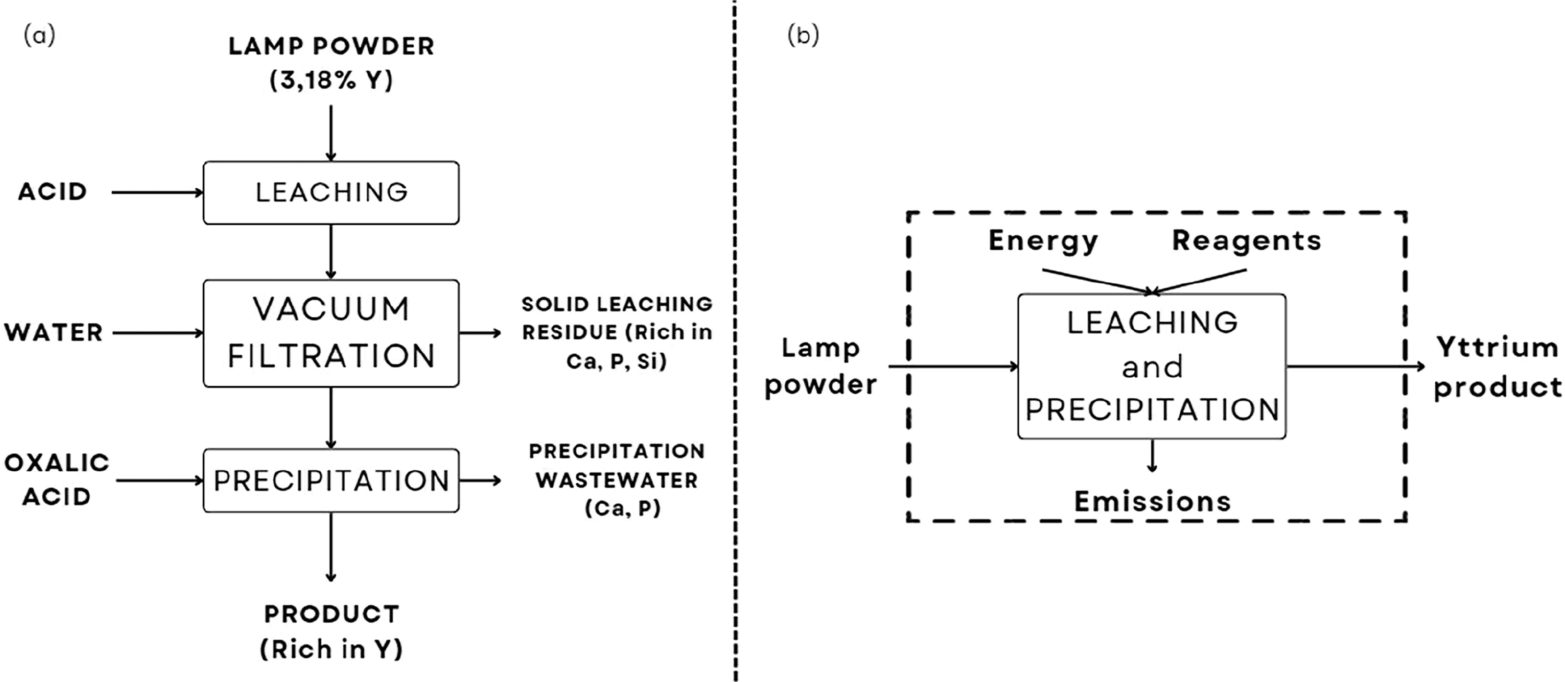
Simplified flowchart of the process for recovery of Y from obsolete fluorescent lamps in a lab scale on the left (a) and system boundary of the process used for LCA calculation with SimaPro 8 software on the right (b).

## 3 Results and Discussion

### 3.1 Characterization

The granulometric analysis allowed concluding that the lamp powder sample is mainly (65%) composed of particles ranging 2–30 μm (Figure S1), with particles ranging 0.3–2 μm (10%) and 30–200 μm (25%).^13^ It was identified that the presence of Ca_10_(PO_4_)_6_F and Y_2_O_3_ (Figure S2); however, it is known that the sample composition (Table 1) also presented an amorphous silica matrix.^13^

**Table 1:** Percentage of the Main Elements Present in the Fluorescent Lamp Powder Used in the Present Study

element	Y	Ca	P	Si	O	F
composition	3.18%	27.65%	12.82%	12.94%	42.10%	1.31%

The spent powder is composed (in wt %) of 3.2% Y, 27.7% Ca, 12.8% P, 42.1% O, 1.3% F, and 12.9% Si, representing 4.0% Y_2_O_3_, 68.3% Ca_10_(PO_4_)_6_F, and 27.7% SiO_2_, similar to what is reported in the literature.^13,15–17^ The lamp powder contains these compounds primarily because Y oxide imparts luminescent properties that enhance the luminous efficiency and color stability. Apatite plays a key role due to its optical properties, efficiently emitting visible light.^18^ Silica acts as a stabilizer in the phosphor matrix, improving the strength and durability of the phosphors and helping to shape the structure of the lamp components.^19^

### 3.2 Acid Leaching Experiments

The effect of the S/L ratio was studied because it directly influences the efficiency of the Y extraction process, affecting the chemical equilibrium of the reaction, dissolution rate, concentration, time, and liquor viscosity. It is important to emphasize that C_2_H_5_NO_2_ and C_6_H_8_O_7_ practically did not leach the phosphate, reinforcing their selectivity during leaching. eqs 2–5 represent each acid reaction with Y_2_O_3_, and Table 2 shows the acid excess based on these equations, acid concentration, and S/L ratio.^20–23^

$$6{{\mathrm{HNO}}_{3}}_{\left(\mathrm{aq}\right)}+Y_{2}{O_{3}}_{\left(s\right)}\to 2Y({\mathrm{NO}}_{3}{{)}_{3}}_{\left(\mathrm{aq}\right)}+3H_{2}O_{\left(l\right)}$$ 2

$$2C_{6}H_{8}{O_{7}}_{\left(\mathrm{aq}\right)}+Y_{2}{O_{3}}_{\left(s\right)}\to 2{\mathrm{YC}}_{6}H_{5}{O_{7}}_{\left(\mathrm{aq}\right)}+3H_{2}O_{\left(l\right)}$$ 3

$$6{\mathrm{NH}}_{2}{\mathrm{CH}}_{2}{\mathrm{COOH}}_{\left(\mathrm{aq}\right)}+Y_{2}{O_{3}}_{\left(s\right)}\to 2Y({\mathrm{NH}}_{2}{\mathrm{CH}}_{2}\mathrm{COO}{{)}_{3}}_{\left(\mathrm{aq}\right)}+3H_{2}O_{\left(l\right)}$$ 4

$$6{\mathrm{CH}}_{3}{\mathrm{COOH}}_{\left(\mathrm{aq}\right)}+Y_{2}{O_{3}}_{\left(s\right)}\to 2Y({\mathrm{CH}}_{3}\mathrm{COO}{{)}_{3}}_{\left(\mathrm{aq}\right)}+3H_{2}O_{\left(l\right)}$$ 5

**Table 2:** Theoretical Values of Acid Excess Required for Leaching Based on the Stoichiometric Reaction, Varying the Type of Acid, and the S/L Ratio

		S/L ratio		
		1/5	1/10	1/20
acid excess (%)	HNO_3_	1865	4734	10,471
	C_6_H_8_O_7_	2711	5522	11,144
	C_2_H_5_NO_2_	832	1765	3629
	C_2_H_4_O_2_	863	2729	6462

The S/L ratios varying 1/5, 1/10, and 1/20 were fixed at 4.0 mol/L HNO_3_, 4.0 mol/L C_6_H_8_O_7_, 2.0 mol/L C_2_H_5_NO_2_, 4.0 mol/L C_2_H_4_O_2_ at 90 °C for 2h. The concentrations were determined with a standard condition of 4.0 mol/L, but C_2_H_5_NO_2_ was limited at 2.0 mol/L due to its solubility (249.9 g/L at 25 °C). The increase of the S/L ratio from 1/5 to 1/20 promotes higher leaching efficiencies due to the amount of acid available for reaction, which is directly related to the dissociation constant (Figure 2a).

**Figure 2:**
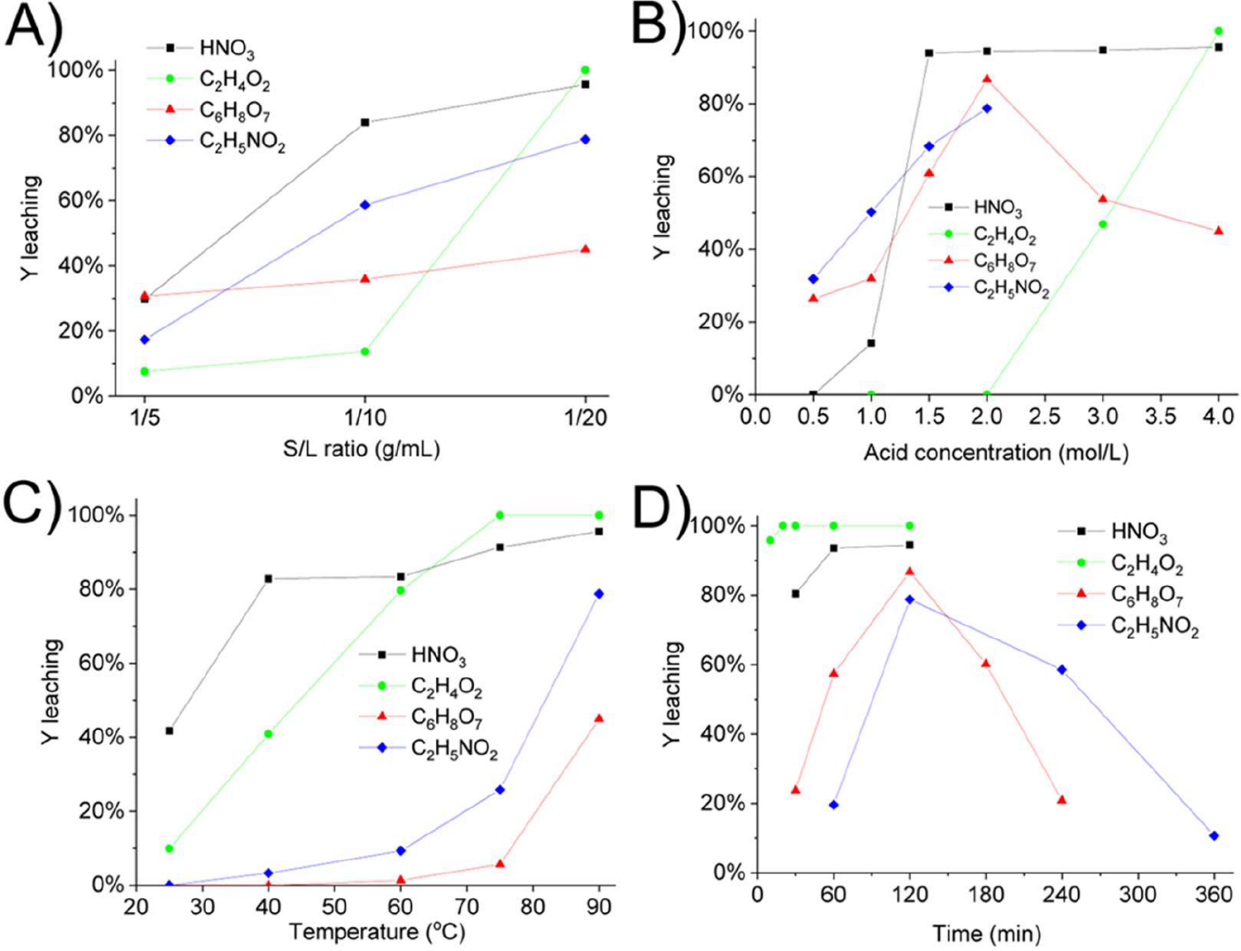
- Percentage of Y extraction varying with the (a) S/L ratio and the type of acid used, at 90 °C for 2 h, and concentration of 4.0 mol/L HNO_3_, 4.0 mol/L C_6_H_8_O_7_, 2.0 mol/L C_2_H_5_NO_2_, and 4.0 mol/L C_2_H_4_O_2_; (b) concentration and type of acid at 90 °C, for 2 h, and S/L ratio 1/20; (c) temperature and type of acid used, S/L ratio 1/20 for 2 h, and concentration of 4.0 mol/L HNO_3_, 4.0 mol/L C_6_H_8_O_7_, 2.0 mol/L C_2_H_5_NO_2_, and 4.0 mol/L C_2_H_4_O_2_; (d) time of leaching and type of acid used, S/L ratio 1/20 at 90 °C, and concentration of 4.0 mol/L HNO_3_, 4.0 mol/L C_6_H_8_O_7_, 2.0 mol/L C_2_H_5_NO_2_, and 4.0 mol/L C_2_H_4_O_2_.

Especially for C_2_H_4_O_2_, the S/L ratio shows a leap from 1/10 (12%) to 1/20 (100%) due to the sharp increase in acid excess. For C_6_H_8_O_7_, it was notable that the S/L ratios studied did not significantly affect Y extraction due to the greater influence of the steric hindrance of the formed complex. Therefore, at the 1/20 ratio, 95.62, 44.95, 78.79, and 100% of Y were leached using HNO_3_, C_6_H_8_O_7_, C_2_H_5_NO_2_, and C_2_H_4_O_2_, respectively.

The excess of acid can compensate for a low ionization constant by increasing the concentration of H^+^ ions for the reaction. In weak acids like C_6_H_8_O_7_ (p*K*_1_ = 3.13; p*K*_2_ = 4.76; p*K*_3_ = 6.40), C_2_H_5_NO_2_ (p*K*_1_ = 2.80; p*K*_2_ = 10.65), and C_2_H_4_O_2_ (p*K*_a_ = 4.76), where ionization is limited, adding more acid improves the leaching efficiency by ensuring a higher availability of reactive species. In the case of strong acids, however, excess acid may not be necessary since the dissociation is already high, optimizing reagent use and preventing waste, as seen with HNO_3_ (p*K*_a_ = −1.4).

The concentration of the acid solution was studied, aimed at determining the acid excess required for Y leaching in a short time period that we believe is feasible at an industrial level (about 2h). The concentration varied from 0.5 to 4.0 mol/L, fixing the parameters at an S/L ratio of 1/20 at 90 °C for 2 h (Figure 2b). It was observed that leaching efficiency was proportional to the increase of acid concentration, reaching 93.9–95.6% using 1.5–4.0 mol/L (HNO_3_). In this case, higher concentrations increase H^+^ in the solution, proportionally impacting the leaching efficiency.

For C_6_H_8_O_7_, the concentration affects the equilibrium reaction linearly, with the acid concentration increasing to 2.0 mol/L (86.7%) and then dropping out. This phenomenon occurs due to the shift in equilibrium and dissociation constant, where an increase in acid concentration can push the reaction toward the formation of more dissociated species (eq 3). However, when the dissociation constant is low, the acid struggles to fully ionize, limiting the availability of reactive H^+^ ions.^24,25^ This negatively affects leaching efficiency by reducing the acid’s ability to effectively break down solid materials and extract valuable components.

For C_2_H_5_NO_2_, a point of maximum leaching (limited at 2.0 mol/L due to its solubility limit) was observed, at 2.0 mol/L (80%), showing higher leaching (0.5–1.5 mol/L) than C_2_H_4_O_2_ and C_6_H_8_O_7_. C_2_H_5_NO_2_ leaching reached 31.9–50.2% (0.5–1.0 mol/L) while nitric-based leaching reached 0–14.2% in the same acid concentration range due to a lower excess of acid. It shows that glycine has potential for Y leaching even at low concentrations and acid excess (Table 3, based on these equations, acid concentration, and S/L ratio). C_2_H_4_O_2_ showed an increased leaching of Y with increasing concentration from 2.0 mol/L (0%) to 4.0 mol/L (100%), as at lower concentrations the leaching process is limited by reaction kinetics. As the concentration increases, the kinetic barrier of activation energy is overcome, causing a sharp change in leaching efficiency.^26^

**Table 3:** Theoretical Values of Acid Excess Required for Lamp Powder Leaching Based on the Stoichiometric Reaction, with Varying the Type of Acid and Concentration

			concentration (mol/L)					
		stoichiometric (mol/L)	0.5	1.0	1.5	2.0	3.0	4.0
acid excess (%)	HNO_3_	0.10	431	1865	3299	4734	7602	10,471
	C_6_H_8_O_7_	0.01	2711	5522	8333	11,144	16,767	22,389
	C_2_H_5_NO_2_	0.05	832	1765	2697	3629	5494	7359
	C_2_H_4_O_2_	0.10	70	863	1796	2,729	4596	6462

The excess of acid significantly increases the leaching efficiency (especially for HNO_3_ and C_6_H_8_O_7_), suggesting a high availability of protons to promote leaching. This can enhance the extraction efficiency at higher concentrations, as more acid is available to react with the target material. However, at very high levels, as observed with C_6_H_8_O_7_, the extreme excess may indicate reagent waste without a proportional increase in efficiency, whereas lower acid concentrations, such as C_2_H_4_O_2_, display a more moderate behavior with incremental efficiency improvements.

The effect of temperature was analyzed as it directly influences the kinetics of chemical reactions and, consequently, the efficiency of leaching. Increasing the temperature typically accelerates reactions, reduces the viscosity of solutions, and increases the solubility of compounds, factors that can optimize the recovery of the metals.^13,27^ The influence of temperature on Y extraction was analyzed from 25 to 90 °C (S/L ratio 1/20, 4.0 mol/L HNO_3_, 4.0 mol/L C_6_H_8_O_7_, 2.0 mol/L C_2_H_5_NO_2_ (limited due to its solubility limit), and 4.0 mol/L C_2_H_4_O_2_ for 2h). The temperature impacted positively on the Y leaching for all acids (Figure 2c), with the maximum at 90 °C to 95.6% (HNO_3_), 44.9% (C_6_H_8_O_7_), 64.8% (C_2_H_5_NO_2_), and 100% (C_2_H_4_O_2_). Y solubility and kinetic energy increase with temperature, promoting higher leaching efficiencies.^25,27,28^

The diffusion mechanism leads to the formation of ion–ion complexes, where smaller Y ions produce relatively simple complexes after the reaction. In contrast, ions with larger radii form bulkier complexes with citric acid (C_6_H_8_O_7_), resulting in reduced diffusion rates and, consequently, lower leaching efficiency. This explains the pronounced differences observed in the acid leaching curves.^27^ Both glycine (C_2_H_5_NO_2_) and citric acid (C_6_H_8_O_7_) form complex ions (eqs 3 and 4), which hinder the leaching process even at elevated temperatures.

The Arrhenius eq (eq 6) is used to analyze the temperature dependence of reaction rates and to extract key kinetic parameters—activation energy (*E*) and frequency factor (*A*) (Table 4) were determined using the linearized Arrhenius eq (eq 7), where *A* is the frequency factor, *E* is the activation energy (J/mol), and *R* is the gas constant (8.31 J/(mol·K)).

**Table 4:** Values of Activation Energies Calculated and Frequency Factor for the Leaching of Y from Lamp Powder for Each Acid Tested

	HNO_3_	C_6_H_8_O_7_	C_2_H_5_NO_2_	C_2_H_4_O_2_
activation energy (J/mol)	9797.7	31,013.8	118,181.8	59,686.20
frequency factor	27.13	3.93 × 10^16^	2.58 × 10^8^	4.15 × 10^8^

To determine the kinetic parameters of Y leaching, the variation of the leaching efficiency with time and temperature was monitored under controlled conditions. The extent of leaching (fraction leached, α, eq 8) was calculated from the ratio of Y leached concentration to the total Y in the solid. Assuming that the process follows apparent first-order kinetics with respect to the solid phase concentration, the rate constant (*k*) was obtained by fitting the experimental α­(*t*) (eq 8). For each acid, *k* was determined at different temperatures (25–90 °C) under a fixed S/L ratio and acid concentration. These *k* values were subsequently used to construct Arrhenius plots (lnk vs 1/*T*, eq 6), from which the activation energy (*E*_a_) and frequency factor (*A*) were determined. Linear regression was applied to obtain *E*_a_ (from the slope) and *A* (from the intercept). Confidence intervals were not included in this study, but goodness-of-fit was evaluated (*R* ^2^ > 0.95). This methodology provides the kinetic constants summarized in Table 4. Fitting results are depicted in Supporting Information (Figure S6). The *k* values were calculated considering eq 7, where lnk was the leaching efficiency and 1/*T* the temperature of the leaching test, and the linear regression produced the values presented in Table 4 and detailed in Figure S6. Expected confidence intervals for the activation energy and frequency factor were not considered in our study.

$$k=A*e^{-E/R.T}$$ 6

$$\ln k=\ln A-\frac{E_{A}}{RT}$$ 7

ln(1−α)=kt 8

High-frequency factor values (Table 4) for C_6_H_8_O_7_ (3.93 × 10^16^) and C_2_H_5_NO_2_ (2.58 × 10^8^) indicate a high theoretical frequency of successful collisions between leaching agents and Y_2_O_3_, which would suggest a favorable reaction path under ideal conditions. However, in practice, factors such as steric hindrance, low dissociation constants, and high activation energies significantly reduce the overall reaction rate, requiring increased temperatures to achieve effective leaching. In contrast, the frequency factor obtained for HNO_3_ was lower (27.13), which may reflect limitations in the fitting process or deviations from ideal Arrhenius behavior. Future experiments may focus on thermodynamic analysis fitting accuracy since it is not the goal of our manuscript, and it is a limitation of our study. Some mechanism change has taken place due to the low k fitting data obtained in our results. This discrepancy, along with the unusually low activation energy value for HNO_3_, suggests that the underlying reaction mechanism may differ substantially from those of the organic acids.

Innocenzi et al. (2017) found an activation energy of 90,300J/mol for Y leaching by H_2_SO_4_+H_2_O_2_,^22^ while Lin et al. (2018) found 61,350 J/mol for Y leaching by H_2_SO_4_ ^29^ which is similar to found by Van Loy et al. (2017) with HNO_3_ (68,000 J/mol for the unmilled and 1,400J/mol for the milled sample).^30^In accordance with Van Loy et al. (2017), different activation energy values were obtained based on mechanical activation varying in a range 10,960–52,820 J/mol as reported by Tan et al. (2017) by HCl leaching.^31^ Our data (Table 4) varies based on the leaching agent used. C_6_H_8_O_7_ has three carboxylic acid groups and one hydroxyl group, allowing it to form strong, multidentate complexes with Y^3^^+^, and these strong interactions require more energy to initiate leaching, increasing the activation energy. C_2_H_5_NO_2_ has both amine and carboxylate groups, forming stable chelates but with less steric hindrance than citric acid—hence moderate activation energy. And C_2_H_4_O_2_ is a small molecule with one carboxylic group and no bulky structure. It forms simpler, weaker complexes with Y^3^^+^, so the reaction requires less energy to proceed.^32^ From the authors’ knowledge, it is the first time these organic acids are being used for the leaching of Y from fluorescent lamps.

The variation in leaching time was investigated as being crucial for optimizing the kinetics of leaching reactions. The contact time between the leaching agent (aqueous phase) and the solid material affects the dissolution rate, influenced by factors such as diffusion and the formation of passivation films.^25^ We aimed at determining the minimum time required for effective leaching, minimizing the use of reagents and energy; for this reason, the evaluated times differ from each other.

Parameters fixed were S/L ratio 1/20, 4.0 mol/L HNO_3_, 4.0 mol/L C_6_H_8_O_7_, 2.0 mol/L C_2_H_5_NO_2_, and 4.0 mol/L C_2_H_4_O_2_ at 90 °C (Figure 2d). The range of time variation was based on results from the literature.^33–35^ C_2_H_4_O_2_ and HNO_3_ had fast kinetic reactions because of their lower activation energy (Table 4). In contrast, C_6_H_8_O_7_ and C_2_H_5_NO_2_ are weak acids and result in a slower leaching rate due to their lower capacity to release hydrogen ions.

HNO_3_ showed an increase in leaching, reaching 100% after 1h, while C_6_H_8_O_7_ (86.7%) and C_2_H_5_NO_2_ (78.8%) reached the peak at 2h. This behavior is related to the kinetics, equilibrium constant, and similarity in the formation of strongly selective complexions, as they only leached Y_2_O_3_ and practically did not react with phosphate. HNO_3_ is a strong monoprotic acid with complete dissociation in aqueous solution (p*K*_a_ ≈ −1.4), providing a high concentration of reactive H^+^ ions that rapidly attack the Y_2_O_3_ matrix, enhancing reaction kinetics and solubility. In contrast, C_6_H_8_O_7_ and C_2_H_5_NO_2_ are weak acids (with p*K*_a_ values of 3.13 and 2.34, respectively) that only partially dissociate, releasing fewer protons and thus requiring more time and thermal energy to achieve comparable leaching levels. Both C_6_H_8_O_7_ and C_2_H_5_NO_2_ form stable coordination complexes with Y^3^^+^ due to their multiple dissociation sites (e.g., carboxyl and amine groups). This complexation is beneficial for selectivity (i.e., limited phosphate leaching) but reduces the rate of metal ion release compared to direct acid attack, as occurs with HNO_3_.

The leaching of C_2_H_4_O_2_ (acetic acid) is noteworthy—it achieved 100% leaching in only 15 min at 25 °C. Despite being a weak acid (p*K*_a_ ≈ 4.76), this efficiency may be explained by a favorable balance of moderate complexation strength and sufficient acidity at high concentration (4.0 mol/L), coupled with its smaller molecular size and lower steric hindrance.^25,27^ For C_2_H_4_O_2_, it can be useful for industrial applications 100% extraction at lower temperatures (25 °C) and shorter reaction time (15 min) compared to other acids studied here.

The pH variation is a critical factor in leaching as it affects the solubility of metals, salt stability, and leaching agent reactivity. Many compounds can precipitate, and equilibrium reactions can shift. It is essential to analyze the influence of pH for C_2_H_5_NO_2_ to ensure maximum process efficiency and effective recovery. For C_2_H_5_NO_2_, the pH of the leach solution was 7.0, while for HNO_3_, it was −1.0. Lower pH values were tested for C_2_H_5_NO_2_ leaching, adding HNO_3_ for adjustment (Figure 3). The pH varied from 0 to 7.0 (S/L ratio 1/20 and 2.0 mol/L at 90 °C for 2h). The choice of the pH range was made starting from pure glycine (pH 7), varying throughout the acidic range on the standard scale from 0 to 14.

**Figure 3:**
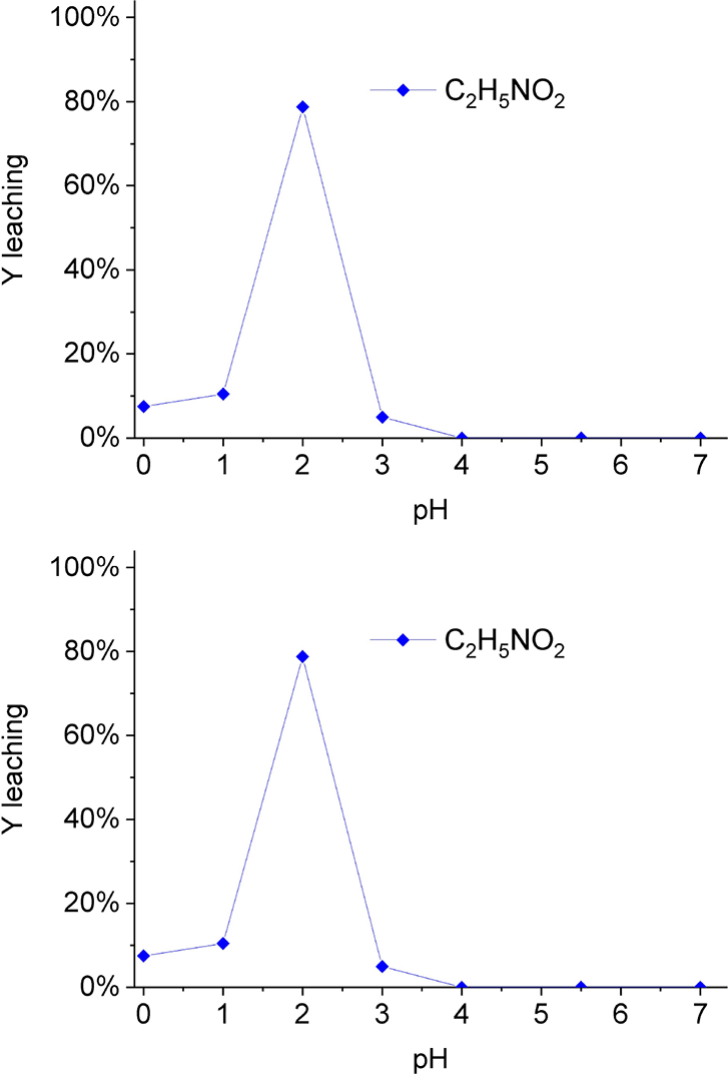
Percentage of Y extraction varying pH of glycine solution, S/L ratio 1/20 for 2 h, and concentration of 2.0 mol/L C_2_H_5_NO_2_.

The influence of the initial pH of the leaching process with C_2_H_5_NO_2_ showed a very pronounced peak in extraction at pH 2.0 that is explained by the formation of a complex ion Y­(GLY)_3_ ^36^ formed from a GLY^+^ and GLY^–^ zwitterion (a hybrid ion that can assume either a positive or negative charge)^36,37^ reaching its acidic equilibrium condition at pH (p*K*_a__COOH_) close to 2.^37^ Therefore, it is crucial to adjust the pH to support the equilibrium reaction and achieve satisfactory extraction.

### 3.3 Comparison of Acid Leaching Reactions

This study evaluated the main parameters influencing the efficiency of Y leaching from spent fluorescent lamp powder using HNO_3_, C_6_H_8_O_7_, C_2_H_5_NO_2_, and C_2_H_4_O_2_. The results allowed for the optimization of operating conditions considering the effects of the S/L ratio, acid concentration, temperature, and reaction time. Table 5 summarizes the leaching efficiencies and parameters

**Table 5:** Example of Leaching Spent Powder for Y Recovery

references	leaching agent	conditions	%Y leaching
current study	HNO_3_	S/L 1/20; 2 mol/L; 90 °C; 2 h	94.5%
Tunsu et al. 2014		S/L 1/10; 0.5 mol/L; 20 °C; 24 h	97%
Botelho Junior et al. 2021	H_2_SO_4_	S/L 1/20; 2 mol/L; 45 °C; 7 h	94%
current study	C_6_H_8_O_7_	S/L 1/20; 2 mol/L; 90 °C; 2 h	86.7%
Prihutami et al. 2020		S/L 1/10; 0.5 mol/L; 45 °C; 4 h	83.35%
current study	C_2_H_5_NO_2_	S/L 1/20; 2 mol/L; 90 °C; 2 h; pH 2	78.8%
Tan et al. 2015	HCl	S/L 1/10; 4 mol/L; 60 °C; 1 h	96.28%
current study	C_2_H_4_O_2_	S/L 1/20; 4 mol/L; 90 °C; 0.5 h; pH 0	100%
Paván et al. 2019		S/L 1/10; 1 mol/L; 20 °C; 10 min	1.25%

The S/L ratio of 1/20 was ideal for maximizing Y leaching, balancing the availability of the leaching agent relative to the amount of residue. However, for C_6_H_8_O_7_, lower excess acid ratios may be explored. One of the most striking results is the selectivity of C_6_H_8_O_7_ and C_2_H_5_NO_2_, which practically did not leach the phosphate from the lamp powder (Figure S3), proving promising for leaching and possible reuse of the leaching residue. Despite that, HNO_3_ and C_2_H_4_O_2_ leaching residues showed an amorphous phase composed of silica (Figure S4).

The experimental results and literature comparisons presented in Table 5 reinforce the critical role of the acid concentration in leaching efficiency. Among the acids tested, HNO_3_ demonstrated the highest Y recovery, achieving 94.5% leaching at 2.0 mol/L, aligning well with literature values such as 97% under milder conditions (0.5 mol/L, 20 °C, 24 h) reported by Tunsu et al. (2014). Citric acid (C_6_H_8_O_7_) and glycine (C_2_H_5_NO_2_), both weak organic acids, reached 86.7 and 78.8% extraction, respectively, at 2.0 mol/L and 90 °C after 2 h. These values are consistent with prior studies using similar weak acids but indicate slower kinetics, likely due to their lower degree of dissociation and strong complexation with Y ions. Notably, acetic acid (C_2_H_4_O_2_), despite also being a weak acid, achieved 100% extraction at 4.0 mol/L and 90 °C within just 30 min. This performance, significantly higher than the 1.25% reported by Paván et al. (2019) at 1 mol/L and 20 °C, highlights the importance of sufficient acid excess and elevated temperature to overcome kinetic barriers in Y leaching.

Raising the temperature significantly accelerated the kinetics of the leaching reactions. For C_2_H_4_O_2_, the temperature increase was particularly advantageous, allowing Y leaching. HNO_3_ (93.6% in 1 h) and C_2_H_4_O_2_ (100% in 15 min) showed maximum efficiency in short times due to lower activation energy, while C_2_H_5_NO_2_ (78.8% in 2 h) and C_6_H_8_O_7_ (86.7% in 2 h) required longer times. However, after 2 h, a drop in efficiency was observed for C_2_H_5_NO_2_ and C_6_H_8_O_7_, suggesting the possible precipitation of Y complexes and thus limiting their effectiveness over extended periods. Additionally, the pH adjustment for C_2_H_5_NO_2_ was crucial for maximizing extraction (78.8% at pH 2.0).

### 3.4 Precipitation of Y Oxalate

Precipitation with H_2_C_2_O_4_ was chosen due to the insolubility of Y_2_(C_2_O_4_)_3_ in acidic solution and its selectivity for Y to obtain the recycling product (eq 9). All the acids studied in leaching were tested under their optimal conditions (Table S2).^38–40^

$$2Y_{\left(\mathrm{aq}\right)}^{3+}+3H_{2}C_{2}{O_{4}}_{\left(\mathrm{aq}\right)}\to Y_{2}(C_{2}O_{4}{{)}_{3}}_{\left(s\right)}+6H_{\left(\mathrm{aq}\right)}^{+}$$ 9

Precipitation with HNO_3_ and C_2_H_4_O_2_ leach solution was not as effective as with C_6_H_8_O_7_ and C_2_H_5_NO_2_ (Figure 4) due to buffering capacities (ability to resist changes in pH) and multiple dissociation sites that allow for more effective interaction with metal ions in solution, reducing the precipitation efficiency. C_6_H_8_O_7_ (citric acid, three carboxylic acid groups and one hydroxyl group) and C_2_H_5_NO_2_ (glycine, one carboxylic acid group and one amine group) have functional groups that can donate protons (H^+^) and form complexes with metal ions like Y^3^^+^.^35–37,41^ Another factor is the complexation and solubility, where these C_2_H_4_O_2_-based complexes can dissociate easily, allowing Y to form Y_2_(C_2_O_4_)_3_ as insoluble precipitate (*K*_sp_ = 5.1 × 10^–30^ in water).^2,41^

**Figure 4:**
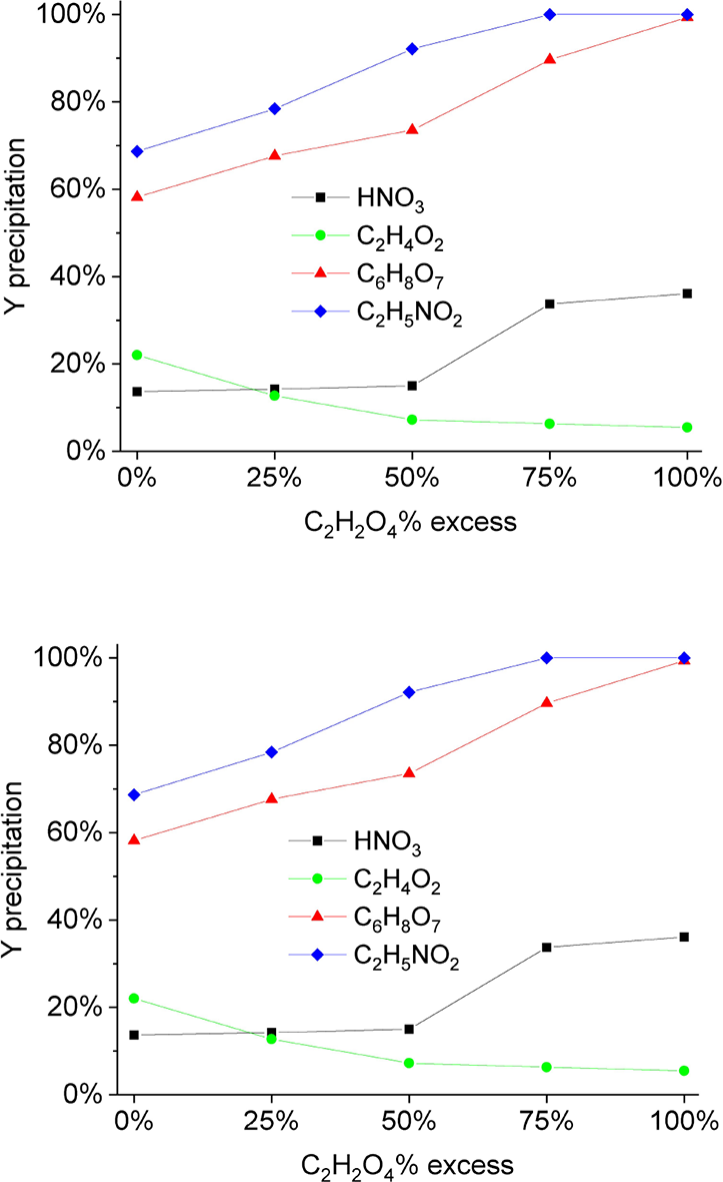
Percentage of Y precipitation varying the stoichiometric excess of oxalic acid and type of acid (from the leaching step). S/L ratio 1/20 at 90 °C for 2 h, and concentration of 2 mol/L HNO_3_, 2.0 mol/L C_6_H_8_O_7_, 2.0 mol/L C_2_H_5_NO_2_, and 4.0 mol/L C_2_H_4_O_2_.

C_2_H_5_NO_2_ and C_6_H_8_O_7_ achieved precipitation efficiencies of 100 and 99.4% (78.8 and 86.2% in global recovery considering leaching and precipitation (Figure 5)), respectively. HNO_3_ and C_2_H_4_O_2_, on the other hand, achieved 36.1 and 5.5% in precipitation (34.1 and 5.5% in global recovery), respectively.^42^

**Figure 5:**
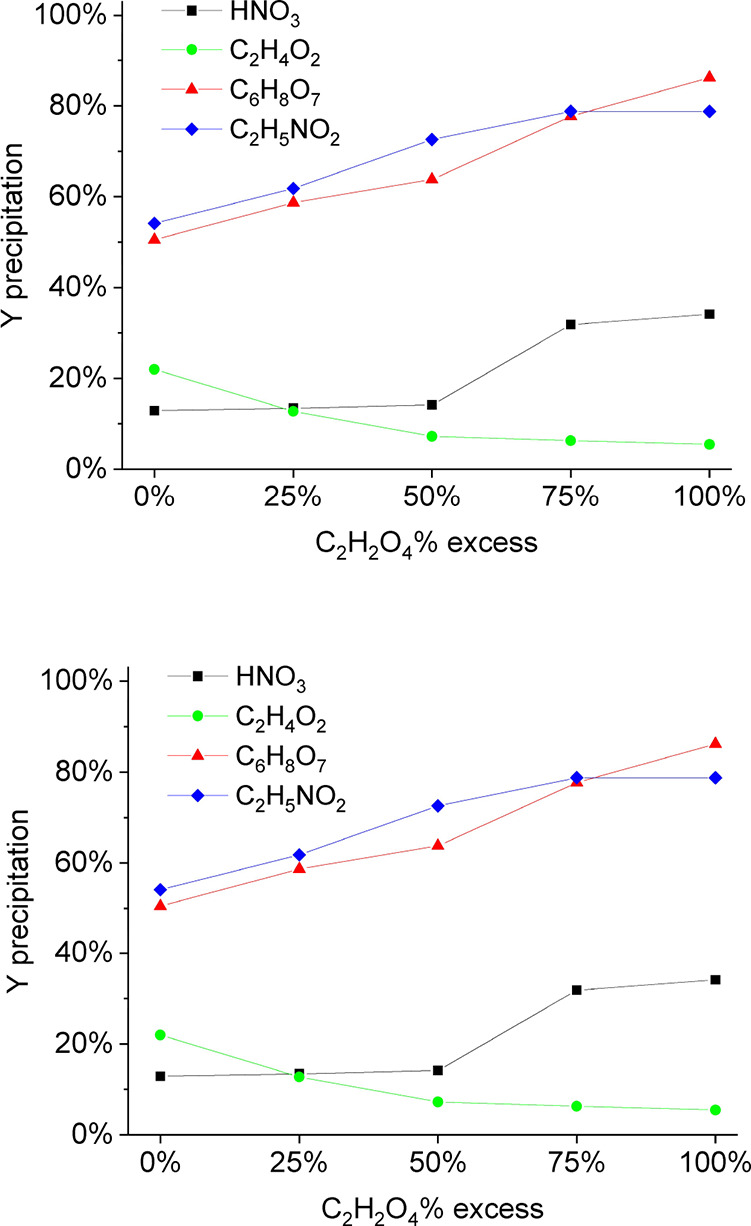
Percentage of Y recovery (leaching + precipitation) after precipitation, varying the excess of oxalic acid and type of acid (from the leaching step). S/L ratio 1/20 at 90 °C for 2 h, and concentration of 2 mol/L HNO_3_, 2.0 mol/L C_6_H_8_O_7_, 2.0 mol/L C_2_H_5_NO_2_, and 4.0 mol/L C_2_H_4_O_2_.

Regarding the matrix of the leaching acid, HNO_3_ affects the solubility and precipitation of the oxalate salt because nitrate anions have a stronger affinity for Y than oxalate anions, and it is highly oxidative.^41,43,44^ C_2_H_4_O_2_ forms acetate salts that have a greater affinity for Y than oxalate.^41,45^ C_6_H_8_O_7_ and C_2_H_5_NO_2_ exhibited similar behaviors due to the complex ions formed (eqs 3 and 4), which are not strong enough to keep the ions in solution when facing a *K*_sp_ of 10^–30^ in water.

Precipitation of C_6_H_2_O_13_Y_2_ from the leach liquor using C_6_H_8_O_7_ and C_8_H_22_N_2_O_23_Y_2_ from the leach liquor using C_2_H_5_NO_2_ was observed (Figure S5). The results align with the literature^46^ and highlight that the precipitated product is indeed an oxalate. For the mass balance (Figure 6), 5g of lamp powder was used, and the best leaching conditions for C_6_H_8_O_7_ (86.7%) and C_2_H_5_NO_2_ (78.8%) were considered. During precipitation, the condition with a 100% excess of C_2_H_2_O_4_ was applied, which resulted in nearly 100% precipitation for both acids.

**Figure 6:**
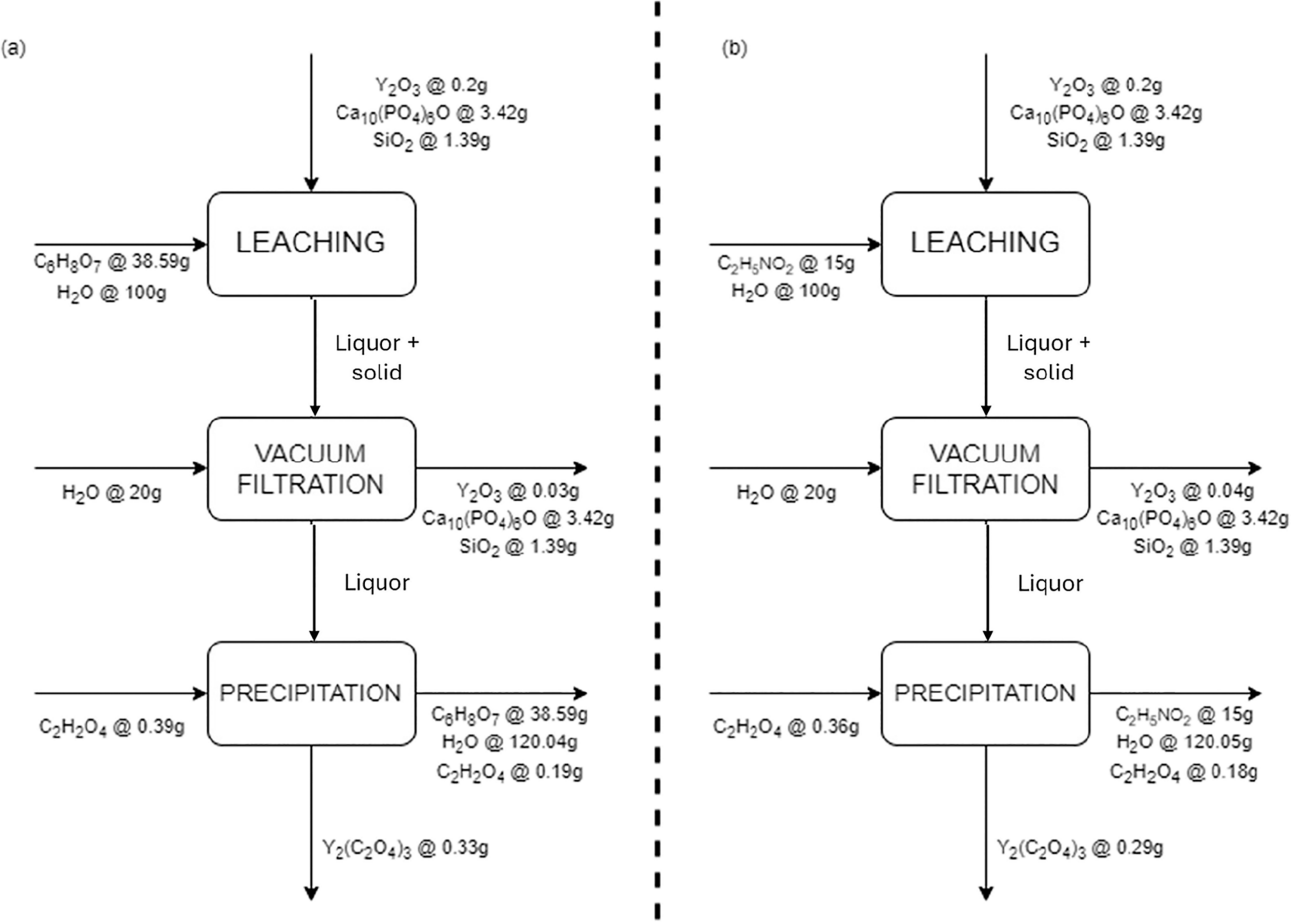
Simplified flowchart of mass balance for recovery of Y from leaching with (a) C_6_H_8_O_7_ and (b) C_2_H_5_NO_2_ under their best conditions and precipitation with C_2_H_2_O_4_.

### 3.5 LCA for Y Recovery from Spent Powder

It is notable that the number of studies on the use of organic acids for hydrometallurgical processes.^11,47–49^ However, most of them are still focused on technical feasibility and just a few from an economic perspective. We proposed a new analysis for Y recovery from secondary sources by LCA to evaluate environmental impacts even at a laboratory scale, comparing organic and inorganic acids. In the present work, the best conditions were used in the leaching stage to compare the impact of the acids used (HNO_3_, C_6_H_8_O_7_, C_2_H_5_NO_2_, and C_2_H_4_O_2_). Our work limitation can be addressed in future investigations that include different energy matrices (wind, solar, hydropower···), water and acid recovery, and scalability. Here, the question to be answered is whether organic acids can have an environmental impact higher or lower than that of mineral acids.

Organic acids are not necessarily more environmentally favorable than inorganic acids (Figure 7) due to the current production path. The most significant contribution is observed in climate change and human toxicity categories, primarily due to reagent production and electricity consumption. A thorough analysis of the process used, scale of operation, and assessment of energy expenditure are required, as these factors have a significant impact on the process. The SimaPro 8 software with the EcoInvent 3.1 database considers a wide range of resources used and effectively analyzes the sustainability of the process, which is crucial for the field of hydrometallurgy.^50–52^

**Figure 7:**
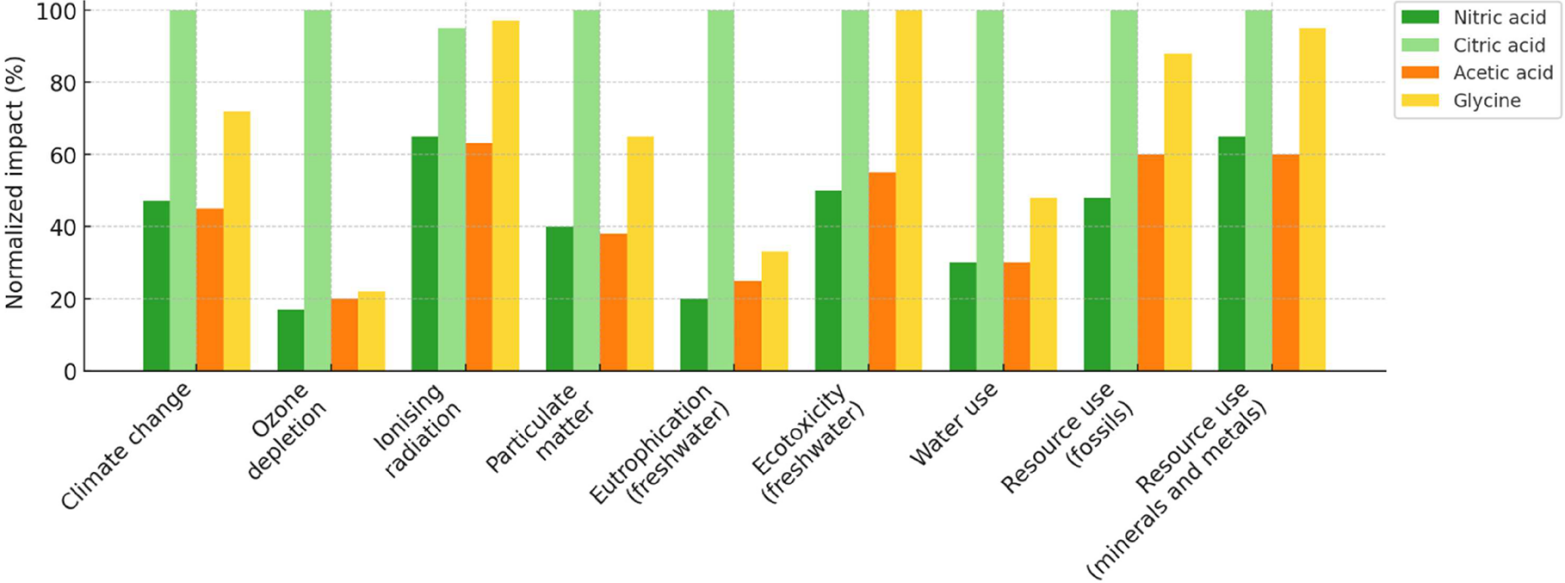
Comparison of the process of recovery of Y using organic and inorganic acids carried out by SimaPro 8.

C_6_H_8_O_7_ and C_2_H_5_NO_2_ have the greatest impact, making it necessary to study more effective routes for their production.^47,49,53,54^ The main impacts of these acids are resource use and ecotoxicity. HNO_3_ and C_2_H_4_O_2_ have similar environmental impacts, indicating that C_2_H_4_O_2_ could be a potential substitute in acid leaching since the price of a liter of glacial acetic acid is generally lower than that of analytical grade nitric acid; on the other hand, Y extraction is technically unfeasible (Figure 7).

Based on the types of impacts associated (*x*-axis), it can be noted that organic acids are not environmentally favorable (Figure 7) as previously supported by the literature.^10^ Their production involves inorganic acids and even more complex processes and reagents. C_6_H_8_O_7_, for example, involves the addition of H_2_SO_4_ and Ca­(OH)_2_ in its production process.^48^ Considering all the energy and resource expenditures for producing organic acids, the environmental impact ends up greater than that of inorganic acids. Overall, the highest impact on the inherent Y recovery process for all acids is energy consumption and mineral or fuel resources associated with the leaching stage and acid production (normalization from Figure 7 by dividing each impact score by the highest value observed within its respective category. This approach scales all values between 0 and 1, enabling the visualization presented in Figure 8).

**Figure 8:**
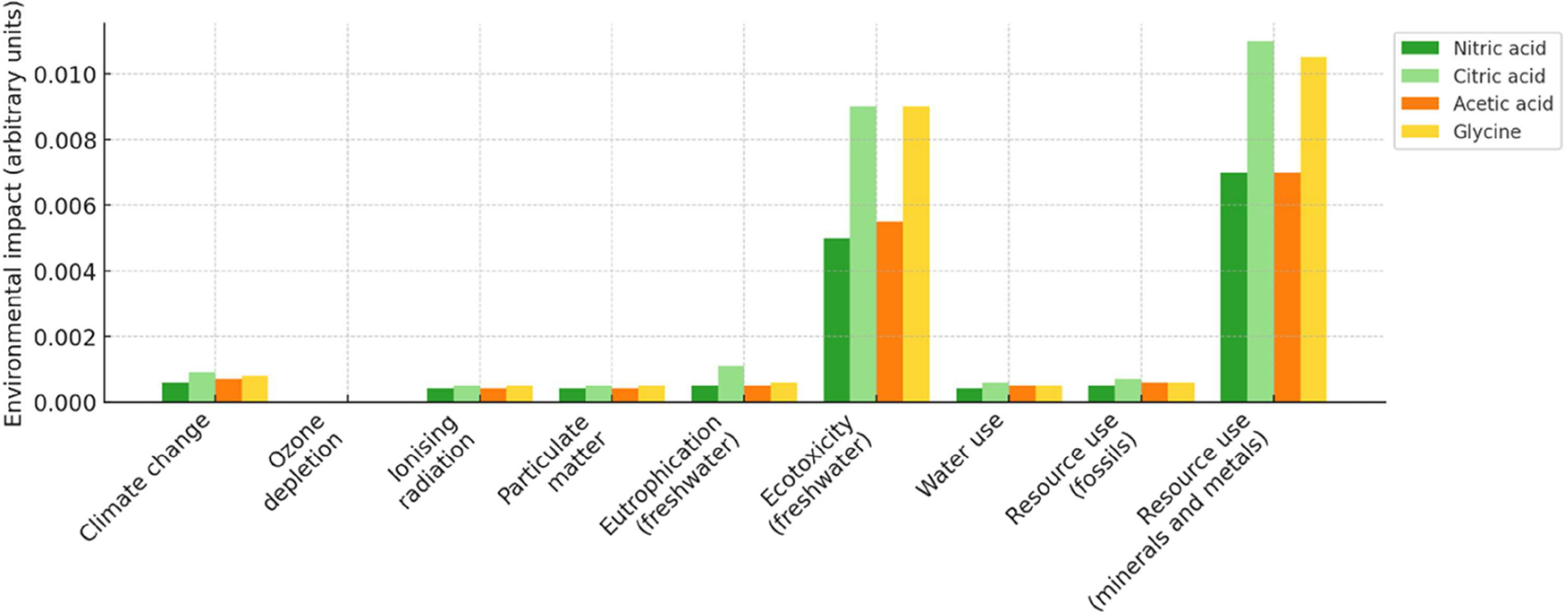
Normalization of the comparison of the Y recovery process using organic and inorganic acids was conducted with SimaPro 8.

High impact associated with resource use and ecotoxicity can be explained by the production stage of organic acids. HNO_3_ has a lower environmental impact than organic acids when comparing the global impact, from both production and final disposal. The impact associated with ecotoxicity (stemming from the treatment of waste) was modeled as “hazardous waste,” indicative of some inherent wastewater toxicity. It is important to consider the treatment of effluents in the disposal of chemical wastes.

The leaching stage is one of the most energy-consuming stages in the process because of the use of high temperature (90 °C). Here, we evaluated these conditions for lab experiments to compare apples to apples. This is evidenced using a condenser with 1370 W of power and heating and stirring plates with 650W of power, both operating for 2 h in the leaching process. An industrial scale will present different results than reported here, and careful analysis is needed for each case. On the other hand, our findings corroborate the literature, and we achieved the goal of comparing inorganic and organic acids for the recovery of Y from spent powder. For future studies, water recovery and challenges for scale-up should be better analyzed; our study focused on the first evaluation of the application of organic acids for a hydrometallurgical process to obtain Y from spent fluorescent lamps.

The LCA results revealed important differences in environmental performance between organic and inorganic acids under the tested conditions. Although organic acids such as citric acid (C_6_H_8_O_7_) and glycine (C_2_H_5_NO_2_) demonstrated high technical selectivity and efficient Y recovery (86.7% and 78.8%, respectively), their environmental impacts were higher across most categories—particularly in resource use, ecotoxicity, and climate change. This is largely attributed to the current industrial routes used for their production, which involve multistep processes, consumption of nonrenewable reagents (e.g., H_2_SO_4_ and Ca­(OH)_2_ for citric acid), and high energy input. On the other hand, HNO_3_, despite its classification as a hazardous chemical, showed a lower overall environmental burden due to its simpler production pathway and higher leaching efficiency achieved at lower acid concentrations and shorter reaction times. Acetic acid (C_2_H_4_O_2_), although achieving 100% leaching, showed limited overall recovery due to a poor precipitation performance and still presented moderate environmental impacts. These findings emphasize that technical performance alone does not equate to environmental sustainability. A careful balance between acid efficiency, selectivity, recovery potential, and upstream production impacts is essential. In future work, scenarios involving acid recycling, renewable energy sources, and biotechnological production of organic acids should be assessed to improve the environmental profile of organic acid–based leaching routes.

## 4 Conclusions

The residue used in our study was a spent fluorescent lamp powder containing 3.18% Y (4% Y_2_O_3_), 68.3% Ca_10_(PO_4_)_6_F, and 27.7% SiO_2_. Furthermore, the best leaching conditions were: HNO_3_ (94.5% leaching) S/L 1/20, 2.0 mol/L, 90 °C for 2h; C_6_H_8_O_7_ (86.7% leaching) S/L 1/20, 2 mol/L, 90 °C for 2 h; C_2_H_5_NO_2_ (78.8% leaching) S/L 1/20, 2 mol/L, 90 °C for 2 h and pH 2; C_2_H_4_O_2_ (100% leaching) S/L 1/20, 4 mol/L, 90 °C for 0.5 h and pH 0. It is essential to analyze the acid excess, dissociation constants, and activation energy of the reactions, as nearly all parameters are greatly influenced by these aspects. C_2_H_4_O_2_ showed the fastest kinetics, being more effective and generating less impact than HNO_3_ under the parameters studied. Otherwise, C_2_H_5_NO_2_ and C_6_H_8_O_7_ exhibited very similar behaviors and demonstrated selective leaching of the material, with practically no leaching of other compounds besides Y from the lamp powder.

For precipitation, C_6_H_8_O_7_ and C_2_H_5_NO_2_ demonstrated superior performance, achieving nearly complete precipitation efficiencies due to their favorable buffering capacities and multiple dissociation sites, which enhance metal ion interactions. HNO_3_ and C_2_H_4_O_2_ were less effective, with lower precipitation and global recovery rates, primarily due to competitive complexation and solubility issues. This highlights the importance of understanding acid-metal interactions and K_sp_ (solubility product constant) to optimize recovery processes.

For preliminary LCA, organic acids are not more environmentally favorable compared to inorganic acids due to their production and disposal impacts, but future scale-up studies can have different conclusions, as our technical approach is feasible, considering also acid and water recovery and biotechnological production of organic acids. While organic acids like C_6_H_8_O_7_ and C_2_H_5_NO_2_ demonstrated good recovery efficiencies, their production pathways and associated environmental impacts, particularly regarding resource use and ecotoxicity, present significant challenges. Inorganic acids such as HNO_3_, although traditionally perceived as more harmful, showed a lower overall environmental impact in this context. The findings emphasize the need for more sustainable production methods for organic acids and careful consideration of the energy and resource demands of the entire process. Further research on scaling up and optimizing acid production is essential to enhance the sustainability of Y recovery in hydrometallurgical processes.

## Supplementary Material



## Notes

Particle size distribution and XRD data of the fluorescent lamp powder; XRD data after leaching by C_2_H_5_NO_2_, C_6_H_8_O_7_, HNO_3_, and C_2_H_4_O_2_; FT-IR analysis of C_6_H_8_O_7_ and C_2_H_5_NO_2_ leaching; kinetic modeling; parameters for leaching experiments; and summary of leaching results (PDF)
