# Supplementary material for: Extraction of Yttrium from Waste: Analysis of Hydrometallurgical Processing by Organic Acids and Life Cycle Assessment
Source: ACS Omega. 2025 Nov 27;10(48):58072–83. doi: 10.1021/acsomega.4c09774 (PMC12771036; doi:10.1021/acsomega.4c09774)
Supplement: Supplementary file 1 [file ao4c09774_si_001.pdf]

# Extraction of yttrium from waste: analysis of hydrometallurgical processing by organic acids and life cycle assessment

Luan Matheus da Silva Alvarenga [1]; Mentore Vaccari [2]; Denise Crocce Romano Espinosa [1]; Amilton Barbosa Botelho Junior *[3,4].

*[1] Department of Chemical Engineering, Polytechnic School, University of São Paulo, São Paulo, Brazil*

*[2] Department Civil, Environmental, Architectural Engineering and Mathematics, University of Brescia, Brescia, Italy*

*[3] Department of Materials Science and Engineering, Massachusetts Institute of Technology, Cambridge, United States*

*[4] Department of Chemical Engineering, Norwegian University of Science and Technology, Trondheim, Norway.*

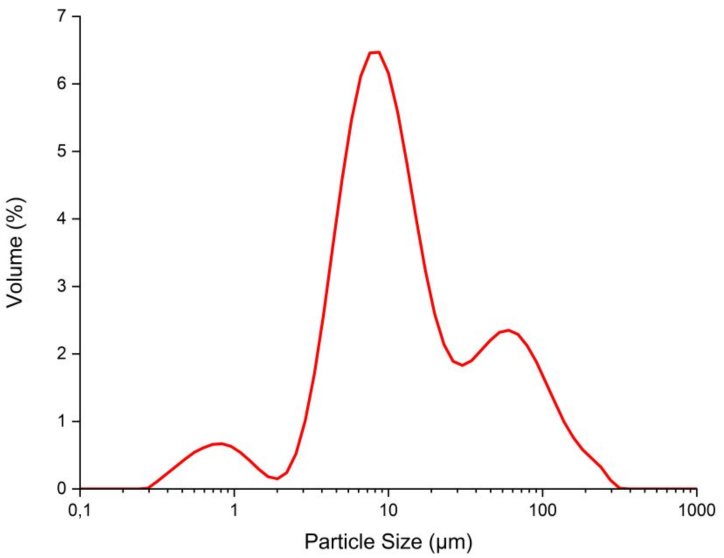


*Figure S1 - Particle size distribution of fluorescent lamp powder.*

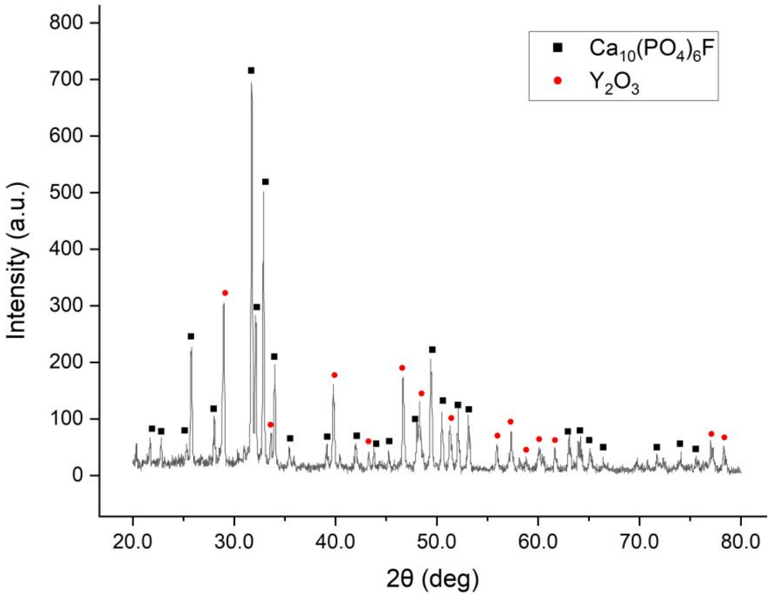


*Figure S2 - Diffractogram obtained in XRD analysis of the fluorescent lamp sample, and the main phases detected.*

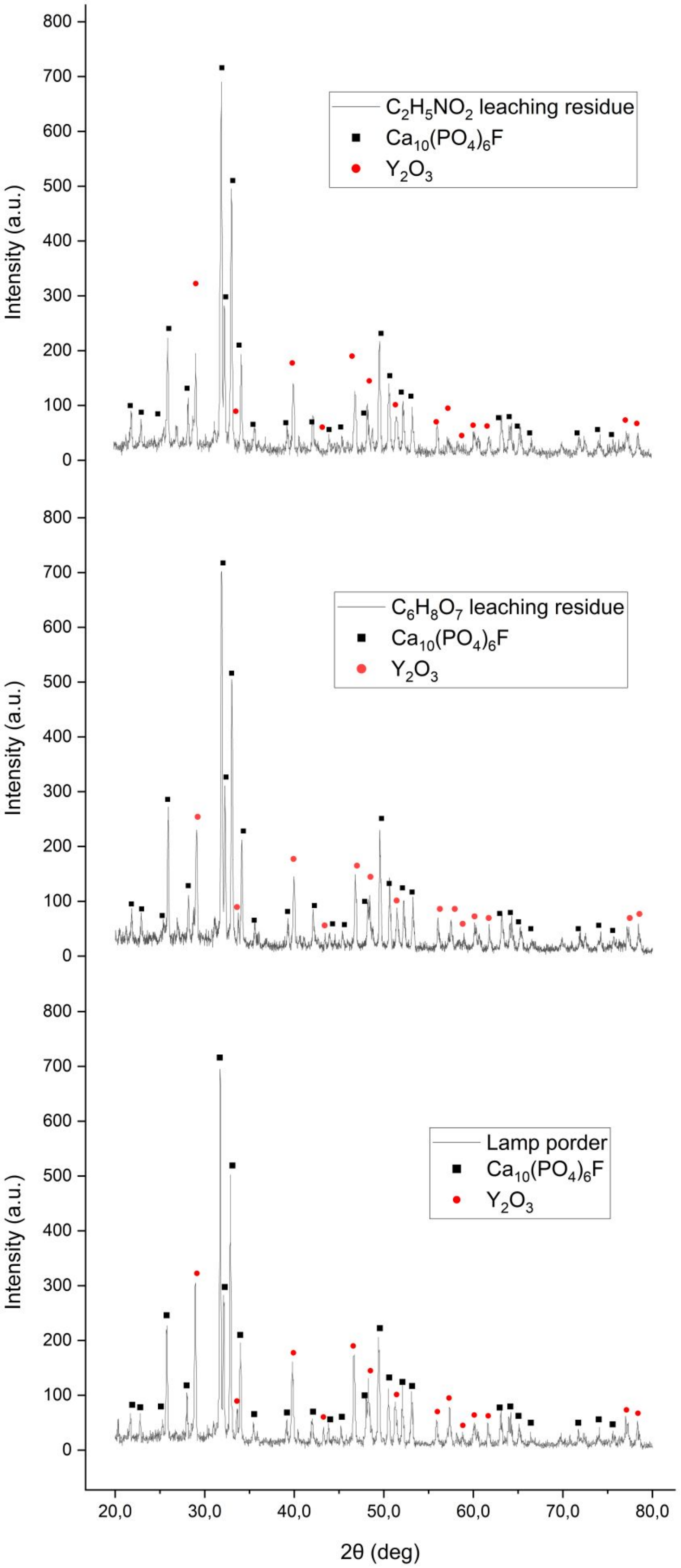


*Figure S3 - XRD of spent powder and for samples leached in the best conditions for $C_2H_5NO_2$ and $C_6H_8O_7$.*

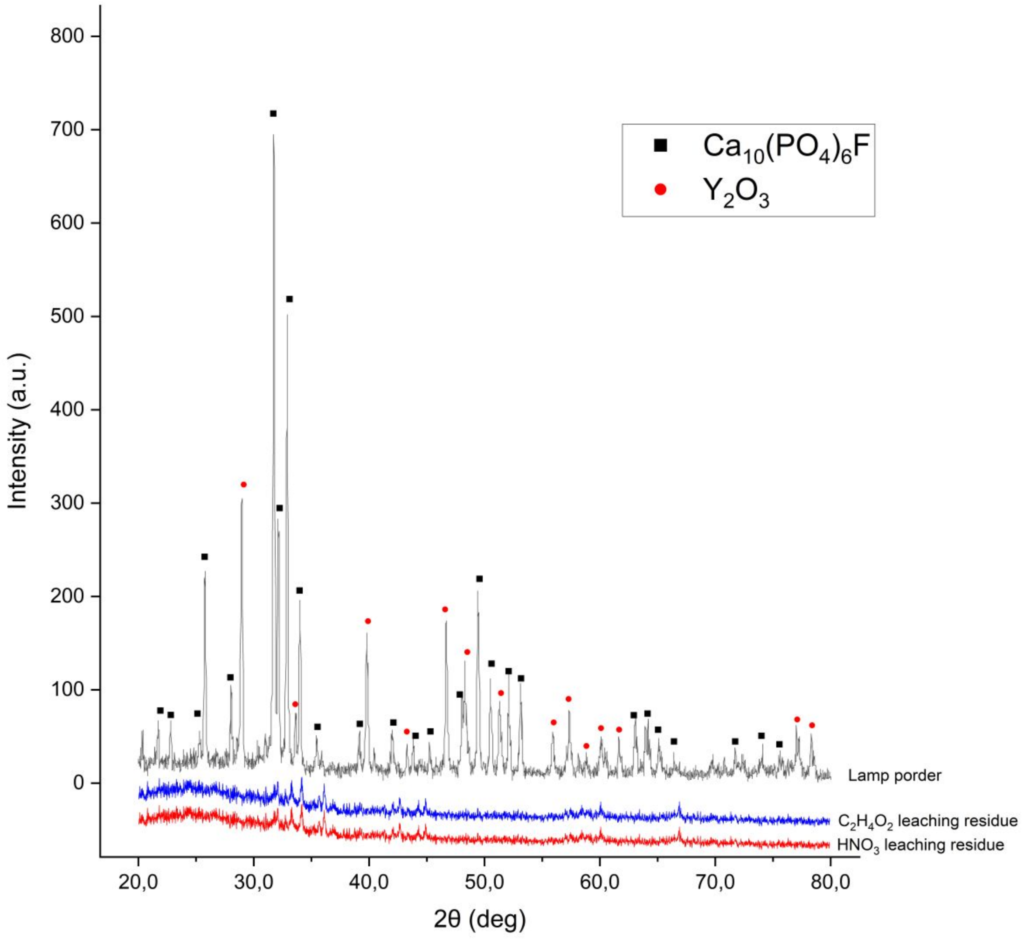


*Figure S4 - XRD of spent powder and for samples leached in the best conditions for $HNO_3$ and $C_2H_4O_2$.*

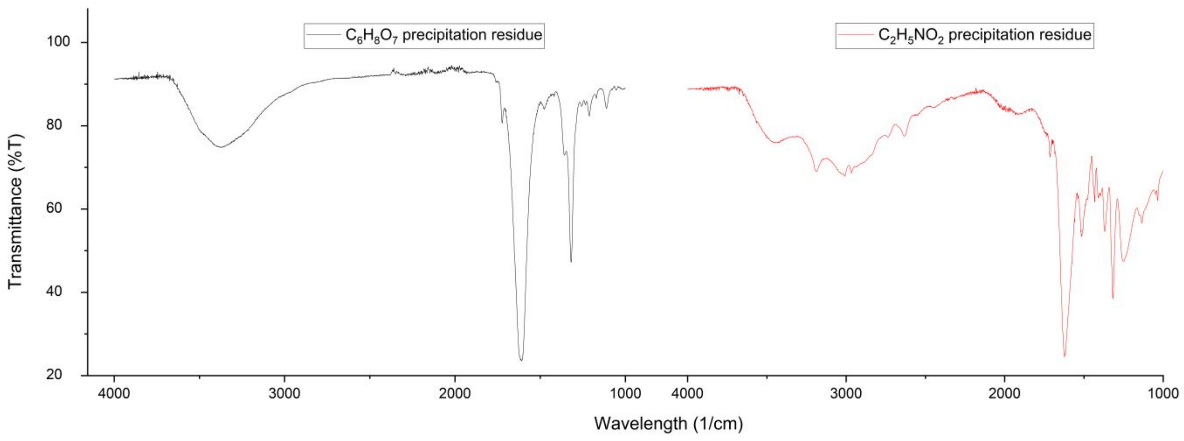


*Figure S5 - FTIR analysis of the precipitation residue from $C_6H_8O_7$ and $C_2H_5NO_2$ leaching.*

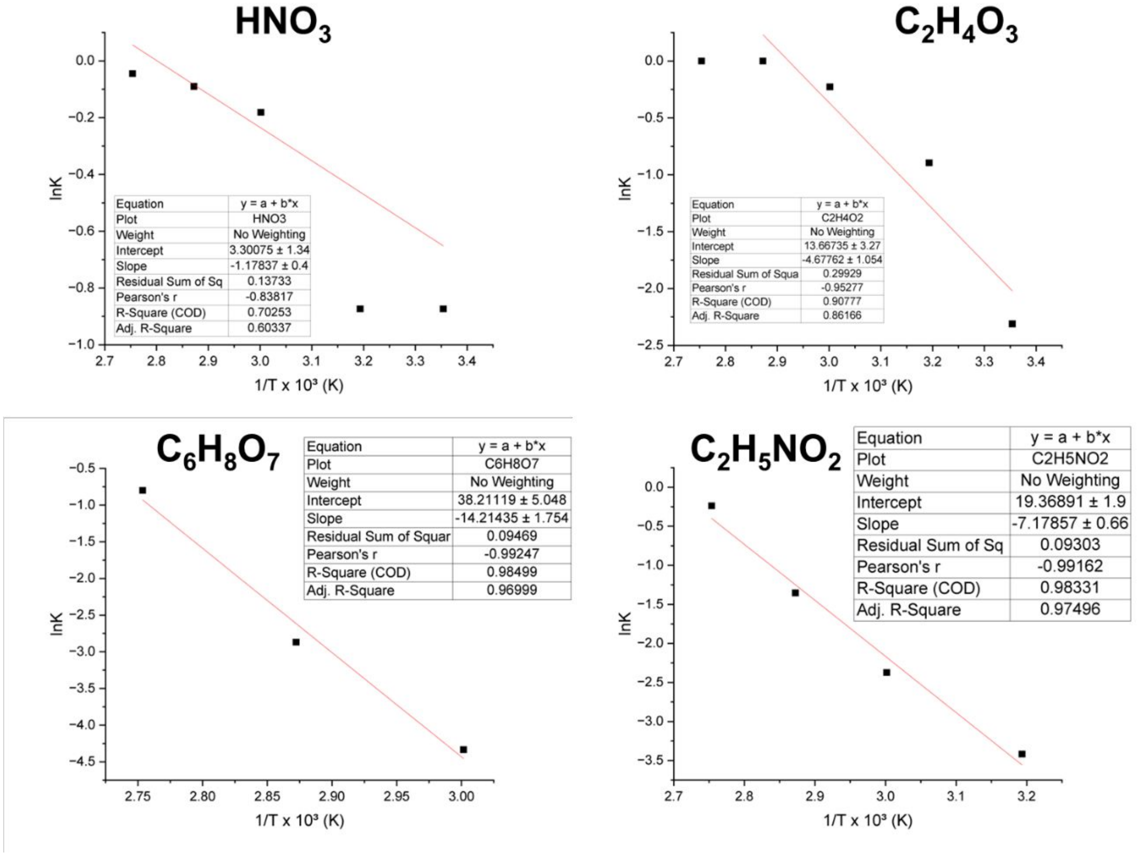


*Figure S6 – Fitting results for the Arrhenius equation calculation*

*Table S1 – Parameters evaluated in leaching using inorganic and organic acids.*

| *Acid* | *S/L ratio* | *Concentration [mol/L]* | *Temperature [°C]* | *Time [min]* | *Initial pH* |
|---|---|---|---|---|---|
| *Nitric ($HNO_3$)* | 1/5<br>1/10<br>1/20 | 0.5<br>1<br>1.5<br>2<br>3<br>4 | 25<br>40<br>60<br>75<br>90 | 30<br>60<br>120 | - |
| *Citric ($C_6H_8O_7$)* | 1/5<br>1/10<br>1/20 | 0.5<br>1<br>1.5<br>2<br>3<br>4 | 25<br>40<br>60<br>75<br>90 | 30<br>60<br>120<br>180 | - |
| *Aminoacetic ($C_2H_5NO_2$)* | 1/5<br>1/10<br>1/20 | 0.5<br>1<br>1.5<br>2 | 25<br>40<br>60<br>75<br>90 | 60<br>120<br>240<br>360 | 0<br>1<br>2<br>3<br>4<br>5.5<br>7 |
| *Acetic ($C_2H_4O_2$)* | 1/5<br>1/10<br>1/20 | 1<br>2<br>3<br>4 | 25<br>40<br>60<br>75<br>90 | 10<br>20<br>30<br>60<br>120 | 0 |

*Table S2 - Leaching conditions of the acids and Y extraction for the precipitation tests with $H_2C_2O_4$.*

| **Acid** | **Conditions** | **[Y] at leach solution (mg/L)** | **Y Leaching (%)** |
|---|---|---|---|
| $HNO_3$ | S/L 1/20; 2mol/L; $90^{o}C$; 2h | 1,502 | 94.5% |
| $C_6H_8O_7$ | S/L 1/20; 2mol/L; $90^{o}C$; 2h | 1,378 | 86.7% |
| $C_2H_5NO_2$ | S/L 1/20; 2mol/L; $90^{o}C$; 2h; pH 2 | 1,253 | 78.8% |
| $C_2H_4O_2$ | S/L 1/20; 4mol/L; $90^{o}C$; 0.5h; pH 0 | 1,590 | 100% |
